# A Comprehensive Review of Acute Type A Aortic Dissection: Epidemiology, Classification, Management Strategies, Mortality Risk Assessment, and Ethical Considerations for Patients who Refuse Blood Transfusions

**DOI:** 10.31083/RCM44307

**Published:** 2025-10-22

**Authors:** Andrei M. Belyaev

**Affiliations:** ^1^Cardiothoracic Surgical Unit, Auckland City Hospital, 2 Park Road, Grafton, 1010 Auckland, New Zealand

**Keywords:** acute type A aortic dissection, taxonomy, socioeconomic deprivation, global cardiac ischaemia, hypothermic circulatory arrest, refusal of blood transfusion, life-threatening anaemia, mortality risk prediction, doctor-patient relationship

## Abstract

Acute type A aortic dissection (ATAAD) is a life-threatening cardiovascular surgical emergency with a mortality of 20–25%. This review offers an overview of current research on the morphology, taxonomy, epidemiology, and anesthetic, perfusion, and surgical strategies involved in ATAAD. Moreover, this review examines methods for predicting mortality risk and explores clinician–patient interactions, particularly those involving patients who refuse blood transfusions. The literature search included PubMed, Google Scholar, Web of Science, and ScienceDirect databases, as well as any relevant books. This review references 144 sources: 129 peer-reviewed articles and 15 book chapters or books. Modern classification systems utilize aortic zones based on the location of intimal tears and the extent of dissection; recent updates have included coronary artery dissection as an additional mapping criterion. Socioeconomic factors are linked to higher ATAAD incidence and poorer long-term survival post-surgery. The duration of global myocardial ischemia correlates with mortality and is a key element in the surgical strategy. Compared to deep hypothermic circulatory arrest (HCA), moderate HCA with cerebral perfusion provides benefits such as reduced bleeding and improved survival. Standard prediction models may not accurately assess risks in patients with life-threatening anemia who refuse blood transfusion. Therefore, incorporating Auckland and Hamilton anemia mortality risk scores alongside conventional tools can improve prognostic accuracy and support personalized management. An interpretive–deliberative model balances patient preferences with surgical outcomes, especially in bloodless surgery. Advances in surgical and endovascular management, as well as postoperative strategies for residual aortic disease, have also been explored. Significant progress has been made in assessing in-hospital mortality, improving doctor–patient communication, refining anesthetic and perfusion techniques, and enhancing surgical management of ATAAD. However, further research is needed to validate these approaches.

## 1. Introduction

Despite significant advances in the prevention and treatment of cardiovascular disease (CVD) over recent decades, CVD remains the leading cause of morbidity and mortality worldwide. In 2021, approximately 20.5 million people died from CVD, accounting for about one-third of all global deaths. Notably, more than three-quarters of these deaths occur in low- and middle-income countries [1].

Acute type A aortic dissection (ATAAD) represents one of the most lethal manifestations within the spectrum of CVDs. The incidence of ATAAD in the United Kingdom is estimated at 50–70 per 1,000,000 population (95% confidence interval (CI): 40–90 per 1,000,000) [2]. Medical management of ATAAD is associated with a high mortality rate of 60–65%, with each hour of delay in surgical intervention increasing the risk of death by approximately 1% [3, 4, 5]. Conversely, emergency surgical repair, while lifesaving, carries its own risk, with mortality rates ranging from 20% to 25% [3].

This comprehensive narrative review offers an overview of current research on ATAAD. This paper begins by examining the morphology and taxonomy of ATAAD, establishing a foundation for understanding its variations and their impact on tailored surgical approaches. It also explores the epidemiology of ATAAD, emphasizing the link between socioeconomic deprivation (SED) and both disease incidence and post-surgical outcomes. The review delves into the pathophysiology of ischaemia, highlighting how modern surgical and perfusion techniques enhance intraoperative management, minimise complications, and boost long-term survival.

Further, it analyses the consequences of blood loss and haemorrhagic shock, critiques the limitations of current in-hospital mortality prediction tools in cardiac surgery for ATAAD patients, and introduces predictive models for life-threatening anaemia in untransfused individuals. This paper emphasizes the importance of effective clinician-patient communication, especially in cases where patients decline blood transfusions. It introduces a novel interpretive-deliberative model that prioritises patient-centred care by balancing respect for patient autonomy with evidence-based medical guidance. Additionally, the review explores innovative approaches in the surgical and endovascular management of extensive ATAAD, as well as postoperative monitoring and management strategies for residual aortic disease.

## 2. Methods

The literature review employed a narrative analysis approach to examine current research on acute type A aortic dissection. The search strategy involved querying multiple databases—PubMed, Google Scholar, and ScienceDirect—using specific keywords including “acute type A aortic dissection”, “hypothermic circulatory arrest”, “anaemia”, “Jehovah’s Witness”, and “doctor-patient relationship”. The inclusion criteria specified that only articles published in English were considered. Additionally, relevant books were also reviewed to supplement the research.

## 3. Results and Discussion

The review references a total of 144 sources, comprising 129 peer-reviewed articles and 15 book chapters or books.

### 3.1 Structural Characteristics of ATAAD

In a typical case of ATAAD, a rupture occurs in the intima, the innermost layer of the aorta, allowing high-pressure blood to enter the media, the middle layer, through a primary tear. This process results in the formation of a false lumen within the aortic wall [6]. During procedures such as coronary angiography or percutaneous coronary intervention, an intimal tear can develop in the coronary arteries, with potential retrograde extension of the false lumen into the aortic root and ascending aorta. In intramural haematoma, bleeding from the vasa vasorum—small vessels supplying the aortic wall—causes media dissection without an initial tear in the intima, although an intimal tear may develop later [4]. The entry point of the tear most commonly occurs in the ascending aorta, but can also be in the aortic arch or proximal descending thoracic aorta. The dissection can propagate antegrade (forward) or retrograde (backward) toward the heart, potentially leading to serious complications such as secondary tears, decompression of the true lumen, or catastrophic rupture resulting in massive bleeding or cardiac tamponade [7].

Involvement of the ascending aorta and aortic root can impair cardiac function by causing acute aortic valve insufficiency, leading to left ventricular failure, or by compressing the coronary ostia, resulting in myocardial ischaemia. Additionally, blood from the false lumen can transudate into the pericardial cavity, causing cardiac tamponade [8].

The dissection may also extend to involve branch vessels, causing either static or dynamic obstruction [9]. Static compression occurs when the intimal flap extends into a branch vessel, narrowing its lumen and impairing blood flow. Dynamic compression involves the overhanging intimal flap during systole, which temporarily restricts blood flow without actual extension into the branch vessel. Both mechanisms can compromise perfusion to the brain, other internal organs, and extremities, leading to ischaemic complications.

Impaired blood flow to internal organs or extremities is observed in 30%–40% of cases of ATAAD type A and is associated with a mortality rate of 45% [10, 11]. Impaired cerebral perfusion occurs in approximately 25% of patients with ATAAD, leading to the development of ischaemic stroke in 47% of patients and a mortality rate of 50% following surgical repair [12, 13, 14].

### 3.2 From DeBakey to the Coronary Arteries: The Evolution and Future of Aortic Dissection Classification

To facilitate effective communication, patient triage, optimal treatment strategies, and outcome assessment in the diverse pathology of acute aortic dissection, M. DeBakey *et al*. [15] proposed an anatomical classification in 1965. The DeBakey classification categorises dissections based on the location of the primary intimal tear and the extent of the false lumen. Types 1 and 2 involve tears in the ascending aorta. Type 1 dissections extend through the ascending aorta, aortic arch, and descending aorta, while Type 2 is confined to the ascending aorta. Type 3 dissections are characterised by a tear and dissection of the descending aorta, distal to the left subclavian artery.

Subsequent data highlighting the efficacy of emergency surgical intervention for ascending aorta and aortic root dissections, coupled with the preference for medical management of descending aortic dissections, led to the introduction of the Stanford classification in 1970 [16, 17]. The Stanford classification focuses on the extent of dissection, without regard to the location of the intimal tear. Type A dissections involve the ascending aorta, whereas type B dissections are limited to the descending aorta. Clinical observations of aortic arch dissections, originating from tears in the arch and descending aorta, have further identified a ‘non-A non-B’ aortic dissection [18, 19].

Modern treatment strategies are largely dictated by the location of the intimal tear, the extent of the dissection, and the presence of organ malperfusion. Consequently, the type, entry, malperfusion (TEM) classification was proposed in 2020, incorporating these factors [20]. Recently, Lombardi *et al*. [21] published reporting standards for aortic dissections established by the Society for Vascular Surgery (SVS) and the Society of Thoracic Surgeons (STS). Unlike the TEM classification, the SVS/STS system utilises 13 zonal segments to delineate the aorta and iliac arteries, based on the presence of the primary intimal tear and the extent of dissection [21].

However, the Stanford, DeBakey, TEM, and SVS/STS classifications of aortic dissection do not account for coronary arteries as the site of the primary intimal tear, with dissection extending into the aortic root and ascending aorta [22, 23]. Furthermore, the National Heart, Lung, and Blood Institute (NHLBI) classification of coronary artery dissection does not include coronary artery-aortic dissections [24]. The NHLBI classification specifically pertains to dissections within the coronary arteries themselves and does not encompass dissections involving the aorta.

Coronary artery-aortic dissections, occurring in approximately six out of every 10,000 percutaneous coronary interventions, are associated with a 20% mortality rate [25, 26]. Therefore, to enhance the taxonomy, accumulate clinical experience, and optimise surgical strategies for ATAAD involving coronary arteries, it is suggested that current classifications be supplemented by incorporating the coronary arteries within the segmental scheme of the aorta and its major branches [22]. While such integration is unlikely to alter the immediate timing of revascularisation—since coronary malperfusion is typically addressed intraoperatively—it could provide valuable guidance for surgical strategy and decision-making.

In conclusion, aortic dissection classification has progressed significantly, from the initial DeBakey and Stanford systems to more recent approaches like TEM and the SVS/STS classifications. However, these systems often fail to address the critical involvement of the coronary arteries. Given the substantial morbidity and mortality associated with coronary artery-aortic dissections, expanding existing classification systems to include this important anatomical consideration is essential. This will facilitate improved communication, more accurate risk stratification, and the development of tailored treatment strategies, ultimately leading to better patient outcomes.

### 3.3 Risk Factors Influencing the Incidence of ATAAD

Epidemiological factors for ATAAD are multifactorial (Table 1). They encompass genetic predispositions, such as connective tissue disorders (e.g., Marfan syndrome and Ehlers-Danlos syndrome), systemic diseases like hypertension and vasculitis, lifestyle factors including smoking and poor blood pressure control, and socioeconomic factors that influence access to healthcare and disease management. Additionally, acquired conditions that weaken the structural integrity of the aortic wall—such as atherosclerosis, trauma, or previous aortic surgery—also contribute to the risk of dissection [27, 28, 29]. Recognising these diverse factors is essential for early diagnosis, prevention, and tailored management strategies.

**Table 1.** **Risk factors for the development of ATAAD**.

Risk factors of acute type A aortic dissection		
Aortic aneurysm (thoracic, thoracoabdominal)		
Atherosclerosis and atherosclerotic ulcers		
Hereditary connective tissue disorders:		
	Marfan syndrome	
	Ehlers-Danlos syndrome	
	Loeys-Dietz syndrome	
Chromosomal disorders:		
	Turner syndrome (45, X)	
Bicuspid aortic valve with aortopathy		
Coarctation of the aorta		
Family history of aortic dissection		
Degenerative changes in the aortic media		
Advanced age		
Smoking		
Use of illicit drugs:		
	Cocaine	
	Amphetamines	
Chronic use of medications:		
	Corticosteroids	
	Immunosuppressive medications	
High socioeconomic deprivation		
Arterial hypertension		
Pregnancy		
Postpartum pituitary infarction (Sheehan’s syndrome)		
Cushing’s syndrome		
Polycystic kidney disease		
Cystic medial degeneration		
Inflammatory and infectious conditions:		
	Large vessel vasculitis:	
		Takayasu arteritis
		Giant cell arteritis
		Behçet’s disease
	Ankylosing spondylitis	
	Aortitis from infections:	
		Bacterial
		Fungal
		Viral
		Spirochetes
		Tuberculosis
Trauma (civilian and military):		
	Blunt trauma	
	Penetrating injuries/Iatrogenic injury	

Despite the undisputed connection between the presence of an aortic aneurysm and aortic dissection, the size of the aneurysm at the aortic root and ascending aorta as a risk factor for ATAAD remains a subject of ongoing debate [30, 31]. According to the clinical guidelines of the American College of Cardiology, candidates for surgical treatment include asymptomatic patients with an intramural haematoma, penetrating atherosclerotic ulcer of the aorta, chronic aortic dissection, degenerative aneurysm of the thoracic aorta, infectious (mycotic) aneurysm of the aorta, or aortic pseudoaneurysm, as well as those with an aortic root or ascending aorta measuring 55 mm or more (Class 1 recommendation, level C evidence) [8]. In patients with genetic connective tissue disorders such as Marfan syndrome, Ehlers-Danlos syndrome, Turner syndrome, and those with a bicuspid aortic valve or a family history of aortic aneurysm or dissection, elective surgical intervention is recommended when the aortic size reaches 40–50 mm (Class 1 recommendation, level C evidence) [8].

These recommendations are based on clinical observations indicating that when the aortic diameter reaches 55–60 mm, the risk of aortic dissection or rupture, as well as death, exceeds the risk associated with planned surgical intervention, which does not exceed five per cent [8]. However, Pape and colleagues [30], using data from the International Registry of Acute Aortic Dissection (IRAD), demonstrated that 347 (60%) of 581 patients with acute aortic syndrome (AAS) had an aortic diameter of less than 55 mm, and in the remaining 40% of patients, the aortic size was less than 50 mm.

### 3.4 The Impact of Socioeconomic Deprivation on ATAAD Risk and Long-Term Surgical Outcomes

The close relationship between socioeconomic factors and health has been known for centuries. Insufficient social support and economic difficulties limit people’s access to food, medicines, and medical care. SED is an important determinant of health and represents a measure of social and economic inequality among individuals or groups in their access to public welfare and resources, including healthcare services and medications. SED acts as a population risk factor, exerting a long-term cumulative impact on individual health. It is associated with adverse outcomes in the treatment of cardiovascular, respiratory, renal, oncological, and other diseases [32, 33, 34, 35, 36, 37].

In a prospective study of 2266 individuals without any clinical manifestations of CVD, Panagiotakos and colleagues [38] found that patients with low socioeconomic status (SES) had an 8% higher systolic blood pressure (*p* < 0.001) and a 4% higher diastolic blood pressure (*p* < 0.001), a 6% higher blood glucose level (*p* < 0.001), and a 7% higher total cholesterol level (*p* < 0.001). Additionally, compared to patients with high SES, those with low SES had a 6% lower level of high-density lipoprotein (HDL) cholesterol (*p* < 0.001), a 22% higher concentration of lipoprotein(a) (*p* < 0.001), an 11% higher level of apolipoprotein B (*p* < 0.001), a 15% higher level of triglycerides (*p* < 0.001), a 45% higher level of C-reactive protein (CRP) (*p* < 0.001), an 8% higher concentration of fibrinogen (*p* < 0.01), and a 7% higher level of leukocytes (*p* < 0.001) [38].

SES is associated with an increased prevalence of arterial hypertension and its complications [39, 40]. In a meta-analysis by Leng and colleagues [41], it was demonstrated that compared to the group with the highest level of education, as an indicator of the highest SES, individuals with the lowest level of education had twice the prevalence of hypertension (odds ratio (OR) = 2.02; 95% CI: 1.55–2.63; *p* < 0.001). In the Antihypertensive and Lipid-Lowering Treatment to Prevent Heart Attack Trial (ALLHAT) study, Shahu and colleagues [39] found that blood pressure control was achieved significantly less frequently among patients from low-income areas compared to those living in high-income areas over the course of 1 and 6 years of the study. The adjusted OR was 0.63 (95% CI: 0.56–0.70; *p* < 0.001) at 1 year and 0.48 (95% CI: 0.37–0.63; *p* < 0.001) at 6 years, respectively [39].

In high-income countries, socioeconomically disadvantaged individuals bear the greatest burden of CVD. Paige and colleagues [42] established that the prevalence rates of absolute risk for a first cardiovascular event were significantly higher among individuals with middle and low SES compared to those in a higher socioeconomic hierarchy—1.4 (95% CI: 1.1–1.9) and 1.6 (95% CI: 1.2–2.2), respectively.

The investigation conducted in New Zealand, which has a universal healthcare system, found that individuals experiencing higher SED have a significantly increased incidence of ATAAD. Specifically, the incidence is approximately 70% higher among individuals who are more socioeconomically deprived compared to those with less deprivation, OR = 1.7 (95% CI: 1.4–2.1; *p* < 0.0005) [43]. During the authors’ study period, 164 out of 363 operated patients (45.2%) died. The overall mortality rate in the cohort of 363 operated patients was 6.9 per 100 person-years (95% CI: 5.9–8.1 per 100 person-years). Of these patients, 74 (45.2%) belonged to the higher SED group, and 90 (54.9%) belonged to the lower SED group (*p* = 0.58). The mortality rate in the higher SED group was 7.6 per 100 person-years (95% CI: 6.0–9.5), while in the lower SED group it was 6.5 per 100 person-years (95% CI: 5.3–8.0). The crude mortality ratio was 0.9 (95% CI: 0.6–1.2; *p* = 0.31). According to the Cox regression analysis, which controlled for differences between groups in age, ethnicity, smoking, and dyslipidaemia, patients in the group with lower SED demonstrated better overall survival. The analysis yielded an OR of 0.7 (95% CI: 0.5–0.99; *p* = 0.045) (unpublished data).

Thus, it can be concluded that SED is associated with an increased incidence of ATAAD and poorer long-term survival among surgical patients following the repair of ATAAD.

### 3.5 Biochemical and Morphological Consequences of Ischaemia: Mechanisms, Pathophysiology, and Cellular Damage

Biochemical and morphological changes that occur during ischaemia are strictly sequential and reflect the severity and duration of blood flow impairment [44]. A decrease in adenosine triphosphate (ATP) levels is the primary cause of ischaemic damage and cell necrosis. In the human body, ATP is produced through two main pathways: oxidative phosphorylation of adenosine diphosphate (ADP) and the glycolytic pathway, which can generate ATP under hypoxic conditions by utilising glucose that enters cells from the extracellular fluid or through glycogen hydrolysis [45].

**Fig. 1.**
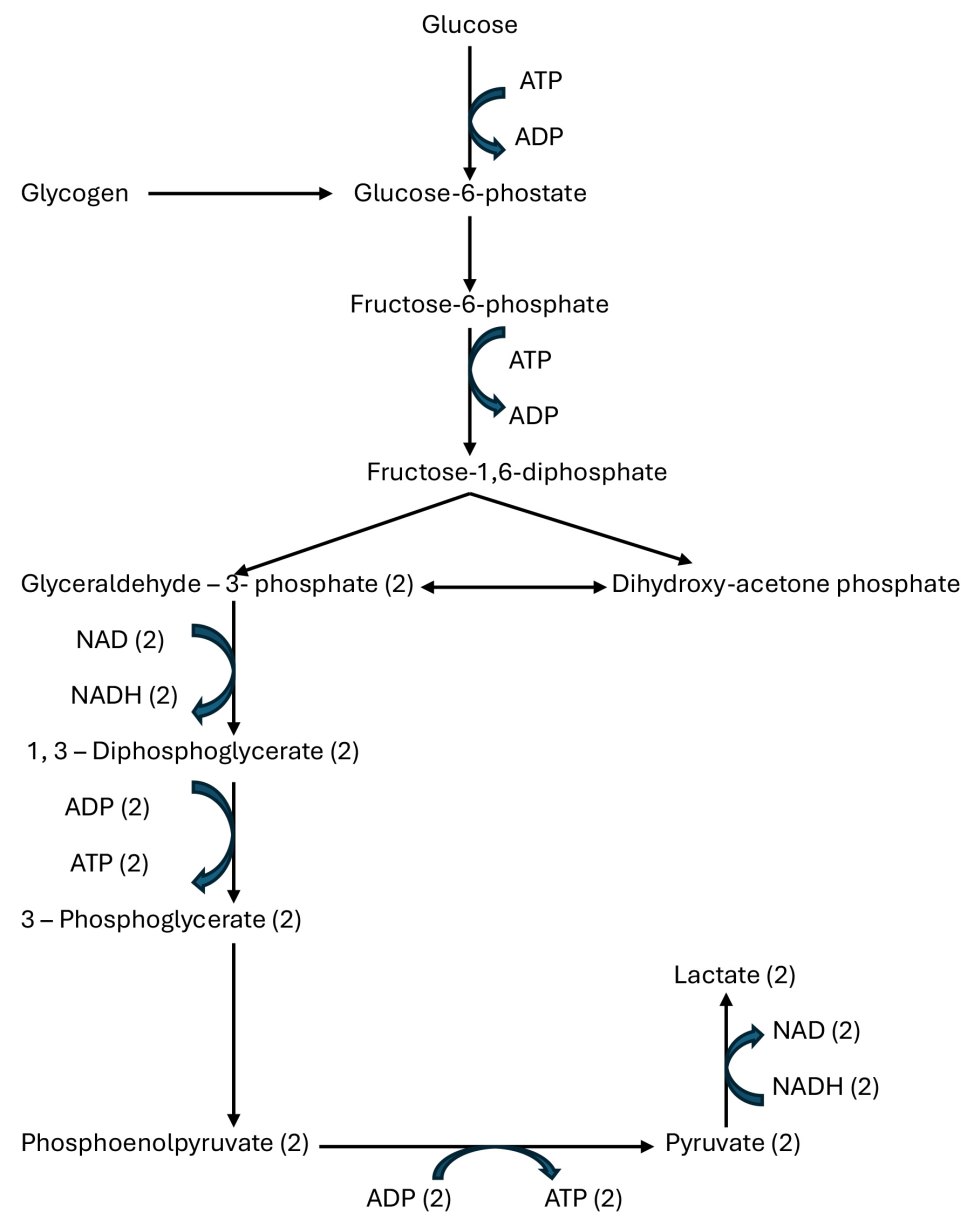
**Glycolysis pathway**. ATP, adenosine triphosphate; ADP, adenosine diphosphate; NAD, an oxidised form of nicotinamide adenine dinucleotide; NADH, a reduced form of nicotinamide adenine dinucleotide.

During glycolysis, the initial investment involves the consumption of two molecules of ATP. Glucose is phosphorylated to glucose-6-phosphate using one ATP (Fig. 1). Then, fructose-6-phosphate is further phosphorylated to fructose-1,6-bisphosphate using another molecule of ATP. This energy investment prepares the molecule for subsequent steps where energy is released and ATP is produced. ATP is produced during glycolysis at two stages: during the cleavage of 1,3-diphosphoglycerate and phosphoenolpyruvate. Since one molecule of glucose yields two molecules of each of these compounds, a total of four molecules of ATP are produced per molecule of glucose during glycolysis [45]. Considering that two molecules of ATP are consumed in the initial reactions of glucose metabolism, the net yield of ATP during glycolysis is two molecules of ATP (the hydrolysis of ATP to ADP and inorganic phosphate releases approximately 7.3 kcal [30.5 kJ] of energy per mole of ATP).

Additionally, during glycolysis, the reduced form of nicotinamide adenine dinucleotide (NADH) is produced, which represents a form of potential energy. If NADH is fully oxidised in the mitochondria, it can yield an additional six molecules of ATP per molecule of glucose. However, for this to occur, NADH must first enter the mitochondria, whose inner membrane is impermeable to NADH. To transport NADH into the mitochondria, an alternative pathway called the ‘malate-aspartate shuttle’ is used. In the first step of this process, the equivalent of NADH reduction is transferred to oxaloacetate, resulting in the formation of malate and the oxidised form of nicotinamide adenine dinucleotide (NAD^+^).

(I) Oxaloacetate + NADH → malate + NAD^+^

Malate produced in the cytoplasm is transported across the mitochondrial membrane, where it is oxidised back to oxaloacetate with the reduction of NADH.

(II) Malate + NAD^+^ → oxaloacetate + NADH

Under the action of the enzyme aspartate aminotransferase, oxaloacetate reacts with glutamate to form aspartate and alpha-ketoglutarate.

(III) Oxaloacetate + glutamate → aspartate + alpha-ketoglutarate

Aspartate and alpha-ketoglutarate exit the mitochondria and return to the cytoplasm, where they can interact again, regenerating oxaloacetate and glutamate.

(IV) Alpha-ketoglutarate + aspartate → oxaloacetate + glutamate

As a result of these conversions, NADH is translocated from the cytoplasm into the mitochondria. However, under hypoxic conditions, oxidative phosphorylation in the mitochondria becomes less efficient, leading to an accumulation of NADH in the mitochondria. This, in turn, causes an increase in the concentration of oxaloacetate and NAD^+^ in the cytoplasm. Since oxaloacetate cannot freely enter the mitochondria, this promotes the accumulation of malate and NADH in the cytoplasm.

Under anaerobic metabolic conditions, NAD is reduced in the reaction of pyruvate with NADH, resulting in the formation of lactate and NAD. This allows cells experiencing hypoxia to regenerate NAD, which is necessary to sustain glycolysis. The lactate produced during anaerobic glycolysis initially accumulates in the cells before entering the bloodstream [46, 47]. When oxygen levels are normalised, lactate can be converted back into pyruvate and subsequently into acetyl-CoA for further participation in aerobic metabolism.

The Krebs cycle is the final common pathway for all oxidative reactions that produce energy. The end product of glycolysis, pyruvate, is transported into the mitochondria, where it is converted into acetyl-CoA. Acetyl-CoA then condenses with oxaloacetate to form citrate, thus initiating the cycle. During the tricarboxylic acid cycle, acetyl-CoA undergoes a series of consecutive transformations, resulting in the formation of reduction equivalents (NADH and the reduced form of flavin adenine dinucleotide, FADH_2_) and guanosine triphosphate (GTP), as well as the release of carbon dioxide [45].

The following equations illustrate these energy-yielding steps:

(V) Pyruvate + NAD^+^ + CoA.SH → acetyl-CoA + NADH + H^+^

(VI) Isocitric acid + NAD^+^ → oxalosuccinic acid + NADH + H^+^

(VII) 2-Oxoglutaric acid + NAD^+^ + CoA.SH → succinyl-CoA.SH

(VIII) Succinyl-CoA + GDP → succinic acid + GTP + CoA.SH

(IX) Succinic acid + FAD → fumaric acid + FADH_2_

(X) Malic acid + NAD^+^ → oxaloacetate + NADH + H^+^

Each molecule of FADH_2_ formed in the Krebs cycle yields two molecules of ATP. In the reaction (VIII), GTP is formed, which is equivalent to one molecule of ATP. In the reaction (IX), one molecule of FADH_2_ is produced. Thus, the breakdown of one molecule of pyruvate in the tricarboxylic acid cycle results in the formation of four molecules of NADH, which are equivalent to 12 molecules of ATP, one molecule of FADH_2_ (2 ATP), and one molecule of GTP (1 ATP). The total number of ATP molecules produced from one molecule of pyruvate is 15. Since one molecule of glucose produces two molecules of pyruvate during glycolysis, this leads to the formation of 30 molecules of ATP in the Krebs cycle, totalling 38 molecules of ATP generated from the breakdown of one molecule of glucose [48].

During β-oxidation, fatty acids are first broken down into acetyl-CoA groups [45]. At each stage of β-oxidation, one molecule of acetyl-CoA and NADH, as well as one reduced flavoprotein (FADH_2_), are formed. Each of these molecules is then used for ATP synthesis in the electron transport chain (ETC). Each acetyl-CoA produced during β-oxidation yields 5 equivalents of ATP. Additionally, when acetyl-CoA passes through the tricarboxylic acid cycle, another 12 molecules of ATP are generated. In the presence of oxygen, fatty acids are efficiently oxidised, providing a high energy yield. However, during hypoxia, fatty acid oxidation does not occur. This is because the necessary enzymes and mechanisms, such as the ETC, cannot function without oxygen.

**Fig. 2.**
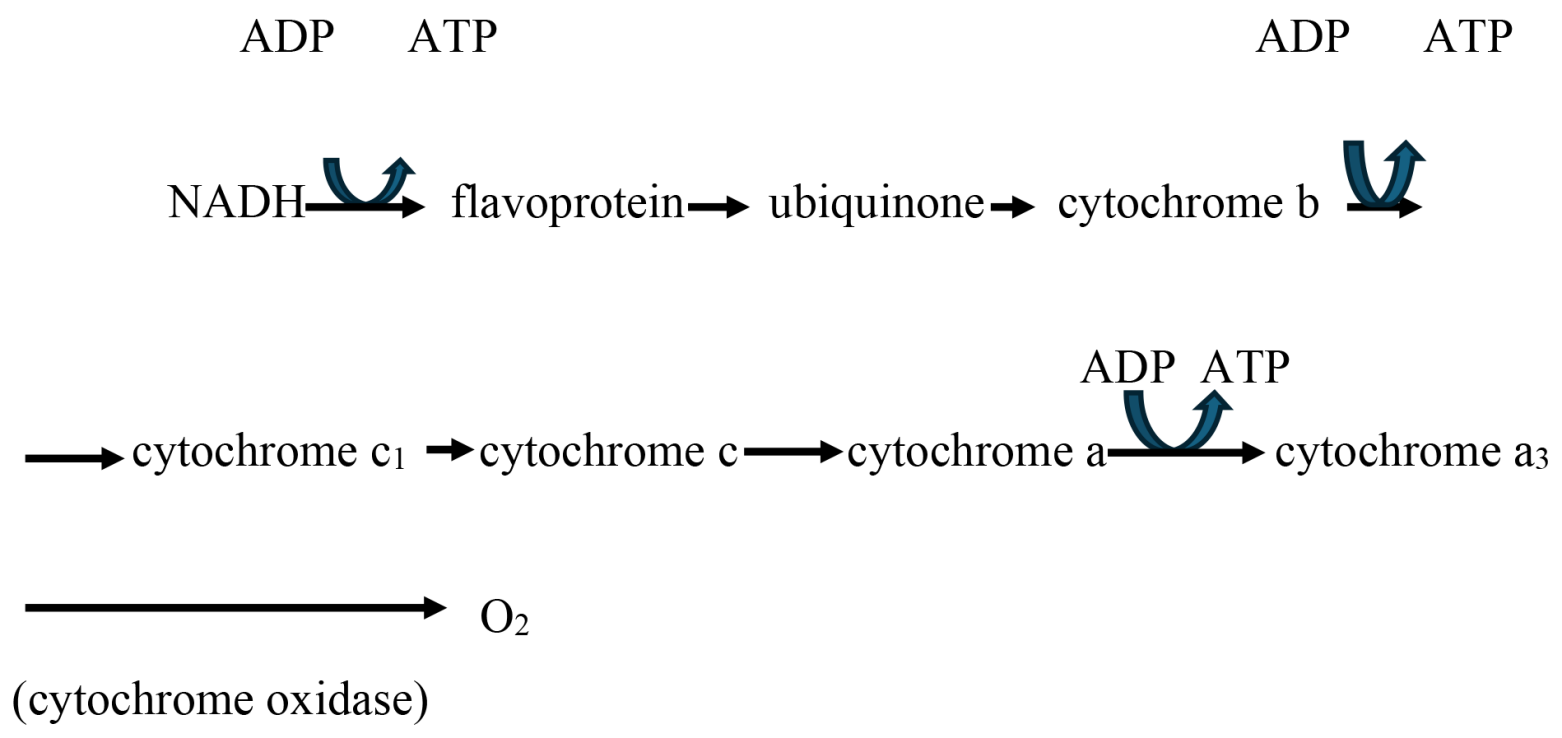
**Electron transport chain**.

During the electron transfer process, which is in the mitochondria, there is a sequential oxidation and reduction of various molecules. This process is accompanied by the release of energy, which is used for the active transport of protons (H^+^) across the inner mitochondrial membrane (Fig. 2). As a result of this transport, a H^+^ gradient (a difference in H^+^ concentration on both sides of the membrane) is created, along with an electrical gradient, since protons carry a positive charge. These gradients represent a form of potential energy, which is then utilised for the synthesis of ATP with the help of the enzyme ATP synthase.

Under hypoxic conditions, the ETC becomes non-functional, causing a halt in oxidative phosphorylation and stopping ATP synthesis. This disruption impairs the activity of the sodium-potassium pump (Na^+^/K^+^-ATPase), leading to the loss of potassium (K^+^) from the cell and the influx of sodium (Na^+^) and water, which results in cellular swelling [49]. The first ultrastructural alteration observed during hypoxic cell injury is the swelling of the endoplasmic reticulum. This is followed by swelling of the mitochondria and overall cellular swelling. During hypoxic depolarisation of the cell membrane, calcium ions (Ca^2+^) also enter the cell [50]. Excess Ca^2+^ can activate various enzymes, including endonucleases, proteases, and phospholipases, which can harm intracellular organelles. As these organelles—such as mitochondria, lysosomes, and cell membranes—begin to rupture, the cell may eventually undergo death via either apoptosis or necrosis [51, 52]. As energy reserves diminish due to hypoxia, glycogen stores are depleted, and protein synthesis declines. Persistent cellular hypoxia worsens the cell’s energy status, causing further structural damage and ultimately leading to cell death.

In hypoxia, the structural components of the cell membrane, such as microtubules and microfilaments, begin to disintegrate [50]. The loss of microvilli from the cell surface results in a decrease in the membrane area available for various cellular functions. The formation of localised protrusions called blebs marks an early stage of cell death and may be accompanied by membrane rupture. During the autophagic degradation of damaged cell membranes, concentric lamellar structures known as ‘myelin figures’ develop, composed of phospholipids derived from the cell membranes and Ca^2+^.

During cellular injury, due to impaired oxidative phosphorylation, protein synthesis, oxidation, and disruption of the ion gradient, mitochondria and the endoplasmic reticulum increase in volume. The concentration of sodium (Na^+^) and chloride (Cl^–^) in the cell rises, while potassium (K^+^) levels decrease. These changes promote water influx into the cell, leading to an overall increase in cell volume.

During tissue reperfusion and the resumption of oxygen supply to the cell, the cell gains the ability to resume oxidative phosphorylation and produce ATP. Many of these changes are reversible, allowing the cell to restore its morphology and functions. However, when ischaemia persists for too long, irreversible cell damage develops, characterised by the destruction of plasma membranes and more pronounced swelling of mitochondria and lysosomes. In the mitochondrial matrix, flocculent amorphous densities form. These can appear as early as 30–40 minutes after the onset of ischaemia, making them early markers of irreversible damage. A massive influx of Ca^2+^ into the cell, especially during reperfusion, can exacerbate cellular injury. Ca^2+^ is involved in the activation of proteases, phospholipases, and other enzymes, which further aggravates the degradation of cellular structures.

Although necrosis is the primary mechanism of ischaemic cell death, hypoxic damage to mitochondria can lead to the release of pro-apoptotic molecules, such as cytochrome c, which promotes the activation of caspases and initiates programmed cell death, or apoptosis.

Upon the restoration of blood flow after a period of ischaemia, pathophysiological processes that exacerbate hypoxic damage occur, ultimately leading to cell death. The main mechanisms contributing to the damage during reperfusion are oxidative stress, excessive Ca^2+^ influx into the cell, and inflammation.

Upon the restoration of blood supply and the influx of oxygen into the cells, the production of reactive oxygen species increases. These free radicals are generated in damaged mitochondria due to incomplete reduction of oxygen, as well as through the activation of oxidases in leukocytes, endothelial cells, and parenchymal cells. Furthermore, under hypoxic conditions, the protective antioxidant mechanisms of the cells are disrupted, leading to the accumulation of free radicals and exacerbating cellular damage [50]. Increased Ca^2+^ influx into the cells begins during the acute phase of ischaemic injury and intensifies during reperfusion. Elevated Ca^2+^ concentrations lead to the formation of pores in the mitochondrial membrane, which in turn disrupts ATP production and exacerbates cellular damage [50].

Ischaemic cell damage can be exacerbated by the development of inflammatory reactions initiated by “danger” signals released from dead cells, as well as cytokines secreted by macrophages. These damage mediators enhance the expression of adhesion molecules on hypoxic parenchymal and endothelial cells, promoting the attraction of neutrophils to the site of injury. Circulating immunoglobulin M and complement deposit on the ischaemically damaged cells, leading to complement activation, which intensifies the inflammatory response and further damages the cells [50, 53].

With advancing age, the cellular capacity to withstand ischaemia-reperfusion injury is markedly reduced. Mitochondrial function declines in aged cells, resulting in decreased synthesis of ATP, elevated production of reactive oxygen species (ROS), and impaired Ca^2+^ homeostasis, which leads to excessive Ca^2+^ influx during ischaemic events [54]. Additionally, endogenous antioxidant defenses, such as superoxide dismutase and glutathione, diminish with age, increasing susceptibility to oxidative stress. Alterations in cell signaling pathways, including reduced activation of Protein kinase B (Akt)/Forkhead box O (FOXO) and Extracellular signal-regulated kinase (ERK) signalling pathways, further compromise the cell’s ability to resist ischaemic injury [55]. Furthermore, aged mitochondria can release mitochondrial damage-associated molecular patterns (mtDAMPs) under stress conditions, provoking inflammatory responses that disrupt cellular homeostasis and impair stress resilience [56]. Collectively, these changes contribute to the increased vulnerability and reduced regenerative capacity of cells following ischaemic episodes.

Thus, in global ischaemia, the extent of ischaemic injury is determined by the duration and severity of the ischaemic episode, and patient-related factors such as age.

### 3.6 Optimizing Surgical Strategies and Safety Measures in the Management of ATAAD

ATAAD is a challenging surgical condition managed using a range of techniques that vary according to the surgeon’s preferences and the policies of different cardiac surgery centres. Some surgeons favour a conservative surgical approach, which involves reconstructing the aortic root with supracoronary replacement of the ascending aorta and hemi-arch replacement [57]. These conservative procedures are generally less technically demanding and can be performed by clinicians with limited experience in aortic arch surgery [58].

Other surgeons prefer a more liberal approach, which, in addition to reconstruction or replacement of the aortic root and the replacement of the ascending aorta, includes partial or complete prosthetic replacement of the aortic arch and its main branches [59, 60, 61, 62]. Operations for the replacement of the aortic arch are more complex than hemi-arch replacement and require longer periods of aortic cross-clamping and cardiopulmonary bypass (CPB). However, proponents of a liberal surgical approach to the aorta in the correction of ATAAD believe that such interventions provide better freedom from reoperations on the aorta and higher 15-year survival rates compared to patients who undergo conservative procedures [60].

Another important element of surgical tactics in ATAAD repair is the consideration of safety limits for the duration of cardioplegic cardiac arrest. Recent findings indicate that longer durations of ischaemia are significantly associated with a higher operative mortality. Specifically, an empirical cut-off point of approximately 150 (95% CI: 126–173) minutes has been identified, beyond which the risk of mortality increases notably (OR = 2.6; 95% CI: 1.5–4.5; *p* = 0.0003) [63]. In cases with shorter ischaemic periods, aortic valve resuspension was performed more frequently, being more than twice as common compared to longer ischaemia cases (OR = 2.5; 95% CI: 1.6–3.9; *p* < 0.00005). Conversely, longer ischaemic durations were associated with a higher likelihood of undergoing Bentall’s procedure, descending thoracic aorta replacement, and concomitant cardiac surgeries, with ORs of 10.9, 4.3, and 4.7, respectively—all of which were statistically significant. Cardiac failure was a more common cause of death among patients with longer ischaemic times, accounting for 10% of deaths compared to 2% in the shorter global cardiac ischaemia group (*p* = 0.001). Furthermore, Cox regression analysis indicated that patients with shorter global cardiac ischaemia durations had better overall survival, with a hazard ratio of 0.6 (95% CI: 0.4–0.8; *p* = 0.002), after adjusting for cerebrovascular disease and urgency of operation [63]. However, it is important to note that the study did not perform subgroup analyses comparing elderly patients to younger patients or those with pre-existing cardiac dysfunction. Consequently, the applicability of the 150-minute threshold across different patient populations remains uncertain. Further research is necessary to determine whether this cutoff should be adjusted based on specific patient characteristics to optimise surgical outcomes.

In conclusion, the management of ATAAD necessitates careful consideration of surgical approaches and operative safety measures. While conservative procedures may be suitable for less complex cases and those with limited surgical experience, more extensive repairs can offer improved long-term outcomes; however, they involve increased operative complexity and risk. Importantly, adherence to safety limits regarding the duration of cardioplegic arrest is crucial, as prolonged ischaemia is associated with higher mortality and adverse outcomes. Therefore, tailoring surgical tactics to incorporate these safety parameters is essential to optimise patient survival and overall treatment success.

### 3.7 Advances in Hypothermic Circulatory Arrest Techniques for Improved Outcomes in ATAAD Surgery

Unintentional hypothermia, combined with acidosis and coagulopathy, is a significant component of the lethal triad of trauma [64]. It hinders the initiation of thrombin generation and the synthesis of fibrinogen, leading to coagulopathy and impaired platelet function. This cumulative effect contributes to increased blood loss and raises the need for blood transfusions [65, 66].

Rajagopalan *et al*. [67] found that even mild hypothermia, defined as a decrease in temperature of less than 1 °C below 35 °C, results in a 16% increase in blood loss and a 22% increase in the relative risk of blood transfusion. Furthermore, there is a consistent association between accidental hypothermia and increased mortality in trauma patients [68, 69].

In cardiac surgery, hypothermic protection is used to reduce cellular metabolism, oxygen consumption, and overall energy expenditure [70]. When treating ATAAD, surgeons often employ the “clamp-off” technique to create a distal anastomosis between the vascular prosthesis and the aorta. This method provides better visualisation of the dissected aorta, facilitates the identification of the primary tear, ensures more accurate suture placement, allows for proper exclusion of the false lumen, and reduces the risk of aortic injury associated with clamping the aorta. The “open distal anastomosis” technique requires the use of hypothermic circulatory arrest (HCA), which provides surgeons with a bloodless operative field and increases the safe time limit for performing the surgery [70].

The ideal temperature for safely performing HCA and utilising additional methods of cerebral perfusion remains a subject of ongoing research and discussion in the current literature [71, 72].

Many experts consider deep HCA (below 20 °C) to be the established gold standard for the surgical treatment of ATAAD. According to a survey conducted in 2015 by Peterson *et al*. [73] among Canadian cardiac surgeons, 54% of respondents indicated a preference for deep HCA [73]. Eight per cent of surgeons preferred HCA with nasopharyngeal temperatures below 18 °C, 46% preferred temperatures ranging from 18 °C to 20 °C, 32% chose temperatures between 21 °C and 24 °C, 12% selected temperatures between 25 °C and 28 °C, while the remaining 2% preferred core temperatures above 28 °C. Many cardiac surgeons utilise additional methods of brain protection during the repair of ATAAD under conditions of HCA, including retrograde and selective unilateral and bilateral antegrade cerebral perfusion. According to the same survey, it was found that 82% of surgeons use cerebral perfusion during ATAAD correction. Specifically, 65% apply only antegrade cerebral perfusion, 10% exclusively use retrograde cerebral perfusion, and 7% employ both antegrade and retrograde methods [73].

Lower temperatures during deep HCA are associated with a higher frequency of reoperation for bleeding and greater volumes of blood transfusion [74, 75]. Recent studies indicate that utilising moderate HCA (MHCA) combined with antegrade cerebral perfusion may offer safety outcomes comparable to or better than deep HCA (DHCA) during surgical repair of ATAAD [75, 76, 77]. Specifically, Belyaev *et al*. (2024) [78] found that patients in the MHCA group experienced lower rates of postoperative complications such as bleeding requiring re-exploration, reduced blood loss, and decreased need for blood transfusions. Additionally, the MHCA group showed fewer instances of renal failure, cardiac arrhythmias, respiratory failure, multiple organ failure, sepsis, and gastrointestinal issues. Importantly, the in-hospital mortality rate was significantly lower in the MHCA group, with an adjusted mortality rate ratio of approximately 0.65, indicating a 35% reduction in mortality compared to the DHCA group. Cox regression analysis further supported this finding, demonstrating a 25% reduction in mortality risk for patients treated with MHCA and antegrade cerebral perfusion [78].

While the evidence indicates that warmer HCA, combined with antegrade cerebral perfusion, may enhance perioperative outcomes and survival in ATAAD repair, the retrospective design of these studies introduces certain limitations. Specifically, the lack of randomisation can lead to uneven distribution of confounding variables between treatment groups, and potential patient selection bias may influence the choice of HCA depth. Additionally, the long study period spanning 20 years encompasses significant advancements in surgical techniques and critical care practices, which could contribute to observed improvements independently of the HCA strategy used. Therefore, these findings should be interpreted with caution, and validation through prospective randomised controlled trials is necessary to definitively establish the benefits of warmer HCA combined with antegrade cerebral perfusion in the surgical repair of ATAAD.

Current evidence suggests that MHCA combined with antegrade cerebral perfusion could offer improved perioperative outcomes and reduced mortality compared to deep DHCA in the repair of ATAAD. While deep hypothermia remains a standard approach, emerging data indicate that warmer temperatures, when combined with effective cerebral protection techniques, can decrease complications such as bleeding, blood transfusions, and organ dysfunction. The retrospective nature of existing studies and the absence of standardised protocols (such as optimal core temperature and cerebral perfusion rates) impede the widespread adoption of MHCA with antegrade cerebral perfusion in the surgical repair of ATAAD. To validate these findings and establish consistent clinical practices, further prospective randomised trials with clearly defined protocols are essential.

### 3.8 Assessing Mortality Risk: Prognostic Models and Risk Factors in ATAAD Surgery

Several risk factors influence in-hospital mortality following surgical repair of ATAAD. These include advanced patient age, female gender, sudden onset of chest pain, hemiparesis, ischaemia of coronary and visceral vessels, diabetes mellitus, atherosclerotic peripheral artery disease, cardiac rhythm disturbances, pulse deficit, critical condition prior to surgery, extension of dissection to the descending thoracic aorta, renal failure, delayed hospital admission and postponement of surgical intervention, recent myocardial infarction, femoral artery cannulation for CPB, and combined surgeries involving the aortic arch [79, 80, 81]. Furthermore, procedural factors such as previous surgeries, recent myocardial infarction, infective endocarditis, low ejection fraction, pulmonary hypertension, emergency procedures, and surgical complexity also impact outcomes.

Various prognostic models have been developed to predict in-hospital mortality. For instance, Mehta and colleagues [80] used indicators such as age, gender, onset of chest pain, pulse deficit, renal failure, shock, and tamponade to create a predictive model. Wen and colleagues [82] proposed using the Acute Physiology and Chronic Health Evaluation (APACHE) II scoring system to forecast in-hospital mortality. The authors found that, compared to patients with ATAAD and an APACHE score of 20 or below, patients with a score between 20 and 25 had more than a tenfold increased risk of in-hospital death (OR = 12.9; 95% CI: 1.7–100.8; *p* = 0.015). Those with an APACHE score above 25 had more than a 90-fold increase in mortality risk (OR = 94.5; 95% CI: 12.6–707.6; *p* < 0.0001) [82].

Czerny *et al*. [83] analysed data from the German Registry for Acute Aortic Dissection (GERAADA) and identified factors such as age, repeat surgeries, hemiparesis, malperfusion, and dissection extension as being associated with mortality. This led to the development of a risk scoring system based on these variables [83]. The final risk prediction model for mortality after surgical repair of ATAAD demonstrated limited discriminatory ability, with an area under the receiver operating characteristic curve (AUC) of 72.5%. Using the coefficients of the variables in the final model, the authors proposed a risk scoring system for mortality after surgical repair of ATAAD based on the GERAADA score [83].

Nashef and colleagues’ extensive prospective study [84] identified several factors associated with increased 30-day postoperative mortality following cardiac surgery. These factors include insulin-dependent diabetes, lung disease, neurological disorders, and preoperative critical status. Additionally, other significant factors linked to in-hospital mortality encompass a history of previous cardiac surgeries, recent myocardial infarction, active infective endocarditis, decreased left ventricular ejection fraction, pulmonary hypertension, emergency procedures, and the complexity of the operation, such as surgeries involving the thoracic aorta. These variables contributed to the development of EuroSCORE II, a prognostic scoring system designed to evaluate operative risk. The study demonstrated that EuroSCORE II has a good discriminatory capacity, with an AUC of 81% in the validation cohort [84]. Based on the study by Ma *et al*. [85], the EuroSCORE II demonstrated a higher predictive ability for 30-day mortality compared to the GERAADA scoring system in a cohort of 1346 patients with ATAAD who underwent surgery in China between 2012 and 2021. Specifically, EuroSCORE II achieved an AUC of 0.708 (95% CI: 0.66–0.79), whereas GERAADA had an AUC of 0.65 (95% CI: 0.61–0.69), with the difference being statistically significant (*p* = 0.002) [85]. However, both scoring systems exhibited limited accuracy, as their AUC values were below 0.75, indicating room for improvement in predictive performance.

In summary, the surgical repair of ATAAD is a complex procedure with the risk of in-hospital mortality. A multitude of factors, encompassing patient demographics, pre-existing conditions, and procedural aspects, contribute to these risks. While several prognostic models, such as those based on the GERAADA score and EuroSCORE II, have been developed to predict mortality, their discriminatory ability remains limited. The available scoring systems, while helpful in stratifying risk, do not currently incorporate factors such as SED or specific clinical challenges like coronary artery involvement and management of patients who refuse blood transfusions. Further research and refinement of risk assessment tools are needed to improve the accuracy of predicting outcomes and guide clinical decision-making in these critical areas of cardiac surgery.

### 3.9 Haemorrhagic Shock: Pathophysiology, Compensatory Mechanisms, and Clinical Management

Shock occurs when there is an insufficient delivery of oxygen and metabolic substrates to tissues and cells, as well as inadequate removal of metabolites, which disrupts oxidative phosphorylation [86]. This disruption affects the ETC in the mitochondria, leading to a reduced production of ATP and an accumulation of metabolic by-products. As a result, cells switch to anaerobic metabolism, which can lead to the production of lactic acid, further contributing to metabolic disturbances [86].

In haemorrhagic shock, the primary event is the loss of circulating blood volume, which triggers a series of physiological responses [47]. Volume receptors located in the atria of the heart respond to the mild reduction in right atrial pressure due to this blood loss. Concurrently, baroreceptors in the aortic arch and carotid bodies, which typically inhibit the autonomic nervous system in response to arterial wall stretch, become activated. However, with significant blood loss, the output from these baroreceptors decreases [87]. This reduction leads to an increased output from the autonomic nervous system, particularly through sympathetic activation at the vasomotor centres in the brainstem. As a result, there is a centrally mediated constriction of peripheral blood vessels, which is an attempt to maintain blood pressure and ensure adequate tissue perfusion despite the reduced blood volume.

The activation of β1-adrenergic receptors results in increased heart rate and contractility, which are essential for enhancing cardiac output. However, this increased workload leads to higher myocardial oxygen consumption [47]. If the oxygen supply to the myocardium is inadequate, it can result in myocardial ischaemia and dysfunction, thereby exacerbating the shock state. Direct sympathetic stimulation of the peripheral circulation, through the activation of α1-adrenergic receptors on arterioles, leads to increased vasoconstriction. This response results in a compensatory rise in systemic vascular resistance and blood pressure. A significant portion of the increase in afterload necessary to maintain mean arterial pressure is achieved through splanchnic vasoconstriction, which redirects blood flow away from less critical organs such as the intestines, kidneys, liver, and skin during shock [88]. In contrast, vital organs like the brain and heart possess autoregulatory mechanisms that strive to maintain their blood flow, even in the context of a global decrease in cardiac output. Additionally, direct sympathetic stimulation causes constriction of veins, which reduces the capacitance of the circulatory system and enhances venous return to the central circulation. This multifaceted response is crucial for preserving blood pressure and ensuring that essential organs receive adequate perfusion during states of shock.

Increased sympathetic output stimulates catecholamine release, primarily epinephrine from the adrenal medulla and norepinephrine from sympathetic nervous system synapses. These catecholamines significantly impact peripheral tissues, enhancing the organism’s response to shock and hypovolemia. They promote hepatic glycogenolysis and gluconeogenesis, increasing circulating glucose availability, stimulate glycogenolysis in skeletal muscle, suppress insulin, and trigger glucagon release. Collectively, these actions boost glucose availability for essential metabolic activities, while hyperglycaemia also serves as an endogenous osmotic load to help retain and increase intravascular volume [47].

Decreased circulating blood volume triggers the hypothalamus to release corticotropin-releasing hormone, leading to the release of adrenocorticotropic hormone (ACTH) by the anterior pituitary. ACTH stimulates the adrenal cortex to release cortisol, which, along with epinephrine and glucagon, induces a catabolic state. Cortisol promotes gluconeogenesis and insulin resistance, resulting in hyperglycaemia, and causes protein breakdown in muscles and lipolysis for gluconeogenesis. Additionally, cortisol promotes sodium and water retention by the kidneys to help restore circulating volume [89].

In response to decreased circulating blood volume, the pituitary gland releases vasopressin (antidiuretic hormone, ADH), triggered by baroreceptors, stretch receptors in the left atrium, and increased plasma osmolality detected by hypothalamic osmoreceptors. Factors such as epinephrine, angiotensin II, pain, and hyperglycaemia further stimulate ADH production. ADH increases water permeability and Na^+^ reabsorption in the distal tubules and collecting ducts of the nephron, thereby reducing water loss and preserving intravascular volume. Additionally, vasopressin acts as a potent mesenteric vasoconstrictor, redirecting blood away from splanchnic organs during hypovolemia, and it works with epinephrine and cortisol to enhance hepatic gluconeogenesis and glycolysis [90].

Haemorrhagic hypovolemia activates the renin-angiotensin system [91]. Decreased renal artery perfusion, β-adrenergic stimulation, and increased Na^+^ concentration in the renal tubules trigger the release of renin from juxtaglomerular cells. Renin converts angiotensinogen to angiotensin I, which is then transformed into angiotensin II by angiotensin-converting enzyme in the lungs. Angiotensin II is a potent vasoconstrictor that affects both splanchnic and peripheral vascular beds and stimulates the secretion of ACTH, ADH, and aldosterone. Aldosterone, produced by the adrenal cortex, promotes Na^+^ and water reabsorption in the nephron in exchange for potassium and hydrogen ions lost in urine. Prolonged renal hypoperfusion can deplete renal ATP stores, resulting in acute renal injury. This condition may manifest as oliguria, anuria, or polyuria [47].

Erythropoietin (EPO) is a glycoprotein composed of 165 amino acids, produced by the endothelial cells of renal capillaries in response to low oxygen levels in anaemia [92, 93]. EPO stimulates erythropoiesis in the bone marrow and restores normal red blood cell counts in the blood over several weeks. It interacts with erythropoietin receptors (EPOR) located on various cell types, including erythroid and non-erythroid cells. EPO binds to EPOR homodimers on early haematopoietic progenitors, such as erythroid burst-forming units, erythroid colony-forming units, proerythroblasts, and basophilic erythroblasts, activating signalling pathways such as Janus kinase 2 (JAK2)/Signal Transducer and Activator of Transcription 5 (STAT5), phosphatidylinositol-3-kinase, Rat Sarcoma (RAS)/Mitogen-Activated Protein (MAP) kinase, and protein kinase C, which promote erythroid differentiation, survival, and proliferation. This leads to an increased production of red blood cells and a reduction in anaemia [94].

In polytrauma patients, haemorrhage often complicates significant soft tissue and bony injuries, leading to the release of “toxins” from injured tissues, referred to as damage-associated molecular patterns (DAMPs) or “danger signals”. In polytrauma patients, haemorrhage often complicates soft tissue injuries, leading to the release of DAMPs from injured tissues. While DAMPs are important intracellular molecules, they become pro-inflammatory mediators when released due to cellular stress or death [95]. Some DAMPs, like extracellular cold-inducible RNA-binding protein (eCIRP), high mobility group box 1 (HMGB1), and histones, are known as chromatin-associated molecular patterns (CAMPs) and come from the nucleus [96]. Extracellular RNA (exRNA) DAMPs mainly consist of micro and ribosomal RNA, along with messenger RNA and cell-free nuclear and mitochondrial DNA [97]. Other DAMPs, such as extracellular ATP and heat shock proteins, originate from the cytosol, triggering a complex state of shock that extends beyond simple haemorrhagic shock [98, 99].

Catecholamines influence immune function by activating pro-inflammatory cytokines. TNF-α, produced by immune cells in response to DAMPs from injured tissues, triggers various physiological responses. These include peripheral vasodilation, the promotion of procoagulant activity, changes in cellular metabolism, and stimulation of other cytokines like IL-1β and IL-6. IL-1β has actions that are like TNF-α and augments the secretion of ACTH, glucocorticoids, and β-endorphins. In conjunction with TNF-α, IL-1β can induce the release of other cytokines, including IL-2, IL-4, IL-6, IL-8, granulocyte-macrophage colony-stimulating factor (GM-CSF), and interferon-γ (IFN-γ) [100].

IL-6 contributes to neutrophil-mediated injury to the lung following haemorrhagic shock and is implicated in the development of diffuse alveolar damage and acute respiratory distress syndrome (ARDS) [101, 102]. IL-6 and IL-1β are mediators of the hepatic acute phase response to injury and enhance the expression and activity of complement, C-reactive protein, fibrinogen, haptoglobin, amyloid A, and α1-antitrypsin. The intensity of complement activation after haemorrhagic shock and trauma correlates with the development of hypotension, metabolic acidosis, coagulopathy, ARDS, and multiple organ dysfunction syndrome [103, 104]. The activation of neutrophils is significantly influenced by cytokines such as IL-6, IL-8, and GM-CSF, which are essential for the immune response. These activated neutrophils generate and release a variety of substances, including reactive oxygen species such as superoxide anion, hydrogen peroxide, and hydroxyl radicals, as well as proteolytic enzymes like elastase and cathepsin G. They can induce lipid peroxidation, inactivate cellular enzymes, and deplete important cellular antioxidants such as glutathione and tocopherol. This release contributes to the systemic activation and adherence of platelets, potentially leading to their clearance from circulation. Such processes can result in microcirculatory thrombosis and regional hypoxia [47].

The vascular endothelium plays a key role in regulating blood flow, facilitating leukocyte adherence, and activating the coagulation system. Surface adhesion molecules—such as intercellular adhesion molecules (ICAMs), vascular cell adhesion molecules (VCAMs), and selectins (E-selectin and P-selectin)—are expressed on endothelial cells and mediate the attachment of leukocytes and platelets to the vessel wall. These interactions enable activated neutrophils to migrate into tissues to combat invading pathogens. However, this process also results in neutrophil-mediated tissue damage through cytotoxic effects, leading to microvascular injury. Such tissue injury can promote microvascular thrombosis, coagulopathy, and organ dysfunction [105].

Shock progresses through three distinct stages: compensated, decompensated, and irreversible, each characterized by specific physiological changes and clinical signs. In compensated shock, the body attempts to maintain adequate blood pressure and organ perfusion despite reduced blood flow. This is achieved by increasing heart rate (tachycardia), constricting blood vessels, and shunting blood to vital organs. Clinical signs include rapid heart rate, rapid breathing (tachypnoea), pale, cool, and clammy skin, narrowed pulse pressure, and altered mental status, such as confusion or anxiety. Importantly, this stage is often reversible with timely intervention aimed at addressing the underlying cause of shock.

When compensatory mechanisms fail, the patient enters decompensated shock. This stage is marked by a significant decline in blood pressure and impaired organ perfusion, leading to end-organ damage. Signs include severe hypotension, weak or absent peripheral pulses, altered mental status ranging from lethargy to unconsciousness, cold and clammy extremities, and evidence of organ dysfunction such as decreased urine output and respiratory distress.

The final stage, irreversible shock, occurs when the body’s compensatory capacity is exhausted, resulting in severe organ damage and cardiovascular collapse that is unresponsive to treatment. This stage carries a high mortality risk and is often associated with disseminated intravascular coagulation (DIC). Clinical features include persistent hypotension despite therapy, multiple organ failure, low or absent urine output, and signs of DIC, such as widespread bleeding and clotting abnormalities.

Since the magnitude of blood loss correlates with the physiological and clinical manifestations in patients, the volume of blood loss can be estimated based on physiological parameters and clinical signs. The American College of Surgeons classifies blood loss into four classes [106]. Loss of less than 15% of circulating blood volume (CBV), approximately equivalent to the volume of blood drawn from donors, constitutes the first class of blood loss. This level of blood loss is associated with only a slight increase in heart rate and does not significantly affect cardiac output, systolic, or pulse blood pressure. Such blood loss in healthy patients does not lead to threatening clinical manifestations and does not require blood transfusion, as the circulating blood volume can be restored within 24 hours due to the influx of extracellular fluid into the vascular space.

The second class of blood loss occurs with a loss of 15% to 30% of CBV and is characterised by tachycardia (heart rate of 100–120 beats per minute), tachypnoea (respiratory rate of 20–30 breaths per minute), and a decrease in pulse pressure associated with an increase in diastolic blood pressure due to elevated levels of circulating catecholamines, which cause increased tone and resistance in peripheral vessels. Other clinical signs associated with this level of blood loss include minor changes in the central nervous system, such as feelings of fear, anxiety, and aggression. In the second class of blood loss, there is also a reduction in urine output to 20–30 mL/hour. Treatment of patients using crystalloid solutions typically leads to stabilisation of their condition; however, some patients in this category may require a blood transfusion.

In patients with a loss of 31% to 40% of CBV, which constitutes the third class of blood loss, classic signs of organ perfusion impairment are observed. These include pronounced tachycardia (heart rate of 120–140 beats per minute) and tachypnoea (respiratory rate of 30–40 breaths per minute), significant changes in consciousness, as well as a decrease in systolic and pulse blood pressure, and oliguria (urine output of 5–15 mL/hour) [107]. Controlling the source of bleeding is the primary treatment goal, which may involve embolisation of the bleeding vessel or emergency surgical haemostasis. Typically, treatment for shock in these patients requires transfusion of allogeneic red blood cells (ARBC) and other blood components.

In the fourth class of blood loss, the volume of blood loss exceeds 40%, which poses a life-threatening situation. Clinical symptoms in such patients include severe tachycardia (heart rate over 140 beats per minute), tachypnoea (respiratory rate over 35 breaths per minute), a significant reduction in systolic blood pressure, and minimal pulse pressure. Additionally, diastolic blood pressure may be so low that it cannot be measured. Some patients may exhibit bradycardia, which can serve as a precursor to impending cardiac arrest and death. These patients often experience decreased body temperature and pallor of the skin, along with significant dysfunction of the central nervous system and pronounced oliguria or anuria. Acute blood loss exceeding 45% of the total blood volume is typically fatal unless patients receive prompt blood transfusions and emergency surgical intervention [108].

Thus, haemorrhagic shock is a critical medical emergency, characterised by insufficient oxygen delivery and metabolic substrate supply to tissues, leading to cellular dysfunction and ultimately organ failure. The body initiates a complex cascade of compensatory mechanisms, including sympathetic activation, hormonal responses, and fluid shifts, to maintain perfusion and restore blood volume. However, these mechanisms can be overwhelmed, leading to the progression through the distinct stages of shock—compensated, decompensated, and irreversible—each marked by worsening physiological derangements and increasing mortality risk. Effective management of haemorrhagic shock requires prompt recognition, rapid control of bleeding, and proactive resuscitation strategies, including fluid and blood product administration. A thorough understanding of the underlying pathophysiology, the stages of shock, and the interplay of various physiological systems is essential for guiding clinical decision-making and improving patient outcomes in this life-threatening condition. Early intervention and a systematic approach are crucial to prevent the progression to irreversible shock and ensure the best possible chance of survival.

### 3.10 Beyond Standard Scores: Utilising Specialised Tools for Mortality Prediction in ATAAD Patients Refusing Blood Transfusion

Considering that up to 15% of patients with ATAAD experience shock, multiple organ failure, and DIC, patients with ATAAD are at an increased risk of massive perioperative bleeding [109, 110]. Surgical repair of ATAAD in HCA also leads to activation of the coagulation system, which exacerbates the course of DIC, increasing postoperative blood loss.

According to Zhang *et al*. [111], massive bleeding during surgical repair of ATAAD complicates the postoperative period in approximately 20% of patients. Postoperative massive blood loss is associated with prolonged aortic clamping, extended CPB time, re-sternotomy to control bleeding, acute renal failure, respiratory failure, stroke, increased hospital mortality, and poorer long-term survival in patients with ATAAD [111, 112]. In a study conducted by McClure *et al*. [113], uncontrolled bleeding ranked as the third leading cause of mortality in patients following surgical treatment of ATAAD, and in more than 20% of those who died, massive bleeding exacerbated the postoperative course but was not the immediate cause of hospital mortality.

In clinical practice, cardiac surgeons encounter ATAAD patients who adhere to the religious beliefs of Jehovah’s Witnesses (JWs) and refuse blood transfusions. Managing untransfused life-threatening anaemia presents a formidable clinical challenge. The data show that patients who refuse transfusions have a markedly higher mortality rate of 20.4%, compared to only 1.9% in those who receive ARBC transfusions [114]. The number needed to treat is 6, indicating that administering ARBC transfusions can save every sixth patient with life-threatening anaemia [114]. Furthermore, the treatment of life-threatening anaemia with low-dose epoetin beta (EPO-β) in these patients did not shorten the duration of anaemia nor reduce mortality, suggesting that this approach lacks clinical efficacy [115].

Effective blood management during surgical procedures necessitates a comprehensive approach that encompasses both Jehovah’s Witness (JW) patient education and the implementation of advanced intraoperative techniques [116]. It is imperative to inform patients about available blood conservation strategies and actively involve them in shared decision-making processes to optimise outcomes. Surgeons must employ meticulous surgical techniques aimed at minimising tissue trauma and intraoperative bleeding, complemented by the application of topical haemostatic agents such as Tisseel, Avitene, and Recothrom, which facilitate local clot formation and reduce bleeding at the surgical site. Additionally, anaesthetic strategies play a crucial role in blood conservation; these include hypotensive anaesthesia, deep sedation, acute haemodilution, utilisation of cell salvage systems, and moderate HCA with cerebral perfusion [78, 116]. Collectively, these measures contribute to a significant reduction in intraoperative blood loss, thereby decreasing the reliance on ARBC transfusions and enhancing overall surgical safety and efficacy.

In JWs presenting with ATAAD, neither the GERAADA score nor the EuroSCORE II is a reliable tool for assessing mortality risk. This limitation arises because these scoring systems often incorporate variables related to transfusion requirements and blood product utilisation, which are inherently restricted or contraindicated in JWs due to their religious beliefs. Consequently, traditional risk stratification models may underestimate or fail to accurately predict operative outcomes in this specific patient population, underscoring the need for alternative assessment strategies tailored to their unique clinical considerations. These mortality prediction instruments must be combined with specialised assessment tools designed to assess in-hospital mortality for patients with untransfused life-threatening anaemia.

Two decades ago, Carson *et al*. [117, 118, 119] demonstrated that in patients with severe untransfused postoperative anaemia the risk of mortality increased significantly—by 2.5 times—for every 10 g/L decrease in haemoglobin concentration, particularly when haemoglobin levels fell below 80 g/L. Despite significant advances in medical care over the past several years, the mortality rate among JWs with life-threatening anaemia remained largely influenced by the lowest haemoglobin level and has not changed significantly [120]. However, nadir haemoglobin concentration alone has been shown to be a poor predictor of in-hospital mortality among severely anaemic JW patients [121]. This indicates that other factors influence patient outcomes in severe untransfused anaemia. For instance, Tobian *et al*. [122] found that in JW patients with life-threatening anaemia (haemoglobin levels below 60 g/L), the presence of conditions such as ongoing bleeding, respiratory failure, renal failure, sepsis, malignant neoplasms, myocardial infarction, cardiac arrhythmia, heart failure, pulmonary embolism, pneumonia, and other infections was associated with increased mortality. The authors also noted that the coexistence of multiple comorbidities further heightened the risk of death in these patients [122].

The Auckland Anaemia Mortality Risk Score (AAMRS) is a tool designed to improve mortality prediction and risk stratification among severely anaemic JW patients [123]. It incorporates various early mortality risk factors, including age (45 years or older), weight (90 kg or more), acute admission, hypertension, cardiac arrhythmia, angina, previous myocardial infarction, valvular heart disease, heart failure, the need for haemodialysis, and haemoglobin level (80 g/L or less on admission). Patients are classified into four risk groups based on their total score: 0–3, 4–5, 6–7, and 8 or above. Corresponding mortality rates increase significantly across these groups, with rates of 4%, 32%, 50%, and 83%, respectively. This scoring system aids clinicians in identifying patients at higher risk of mortality, facilitating more tailored and informed management strategies [123].

The Hamilton Anaemia Mortality Risk Score (HAMRS) is a tool designed to monitor the clinical course of untransfused, life-threatening anaemia and to adjust mortality risk estimates provided by the AAMRS system [124]. It is calculated by assigning points to seven specific anaemia-related risk factors: shock (3 points), acute gastrointestinal bleeding (2 points), pneumonia (2 points), a nadir haemoglobin concentration of 70 g/L (1 point), septicaemia (1 point), worsened congestive heart failure (1 point), and neurological complications such as stroke and hypoxic ischaemic encephalopathy (1 point). The mortality rates associated with HAMRS scores are as follows: patients with scores of 0 to 2 had a 4% mortality rate; scores of 3 to 4 corresponded to a 29% mortality rate; a score of 5 was associated with a 40% mortality rate; and a score of 6 indicated a 67% mortality rate [124].

The surgical management of ATAAD presents significant challenges, particularly when patients adhere to religious beliefs that preclude blood transfusions. While established risk prediction instruments like GERAADA and EuroSCORE II offer valuable insights, they are insufficient for accurately assessing mortality risk in this specific patient population experiencing untransfused, life-threatening anaemia. The data underscore the critical need for specialised assessment tools, such as the Auckland and Hamilton Anaemia Mortality Risk Scores, which are designed to incorporate factors unique to this clinical scenario. By integrating these specialised tools into the risk stratification process, clinicians can gain a more comprehensive understanding of individual patient risk, enable more informed decision-making, and potentially improve outcomes. The use of these tools represents a crucial step towards optimising the management of ATAAD patients who refuse blood transfusions, ultimately aiming to mitigate the heightened mortality risk associated with this complex clinical challenge.

### 3.11 The Interpretive-Deliberative Model: A Framework for Difficult Medical Decisions

In modern medicine, the relationship between the physician and the patient is centred around the rights and needs of the patient. Patients have taken on the role of equal partners and being fully informed about the risks and benefits of diagnostic procedures and treatments; they have the right to make autonomous decisions. This upholds one of the fundamental ethical principles: “respect for patient autonomy” [125].

According to Emanuel EJ and Emanuel LL [126], there are five ethical models of the physician-patient relationship: instrumental, paternalistic, informative, interpretive, and deliberative models. The instrumental model is rejected by physicians on moral grounds, as it does not consider the subjective choices and values of the patient; instead, the physician prescribes treatment based on external objective values, such as social or scientific good. However, this model remains relevant and serves as a warning against the unchecked use of artificial intelligence (AI), where AI is employed not for the benefit of the patient but for efficiency and cost-saving purposes. In such cases, the relationship between the physician and patient may become instrumentalized [127].

The paternalistic model assumes the existence of objective values that allow for the formulation of an optimal treatment strategy aimed at achieving the best possible health outcomes. In this model, the physician acts as a guardian or expert whose role is to promote the well-being of the patient, without considering the patient’s beliefs and preferences regarding their own health. The physician is granted most of the decision-making authority in favour of preserving the patient’s health. Autonomy is realised only through the patient’s agreement with the physician’s recommendations.

The informational model grants the patient a greater share of decision-making authority. This model assumes that the patient is aware of their values but lacks only medical facts. The physician’s role is to provide medical facts, including the benefits, risks, and costs associated with treatment, which will help the patient make an informed decision that aligns with their values and ensures the implementation of the chosen treatment. According to this model, there is no place for the physician to understand the patient’s values or to compare them with their own. This model does not imply a compassionate approach from the physician towards the patient, nor does it involve the physician providing recommendations for selecting the best treatment.

The interpretive model involves a more active role for the physician in understanding the patient’s subjective beliefs and preferences regarding their health. Once the clinician becomes familiar with these treatment-related values, they do not pass judgment but rather acknowledge and validate them. The physician then thoroughly explains all available diagnostic and treatment options that can help realize these values. Ultimately, the decision to choose or refuse treatment rests entirely with the patient. In this model, the physician acts as a consultant or advisor. The primary goal of treatment is to align with the patient’s beliefs and preferences regarding their health, even if that choice contradicts their own interests and well-being. Coercion into medical intervention against the will of a mentally competent patient is considered a criminal offense.

The deliberative model assigns a greater role to the physician not only in assessing the patient’s beliefs and preferences regarding their health but also in prioritising those values to achieve the best treatment outcomes. The physician is expected to help the patient clarify the connection between specific preferences and beliefs about their health and the possibility of achieving the desired treatment results, indicating why certain health-related values are more significant and should be pursued. The goal of this discussion is moral persuasion rather than coercion, with the final determination of which values take precedence remaining with the patient. In the deliberative model, the physician acts as a teacher or friend, engaging the patient in reflection and identification of priority health-related values to develop a personalised treatment strategy aimed at achieving the desired outcome for the patient. During this discussion, the physician employs normative reasoning and persuasion while also considering alternative health-related values, their significance, and implications for the patient. This model views patient autonomy as a tool for moral self-development.

The shortcomings of these models include their failure to account for the mental competence of the patient, as well as the acute psychological regression (APR) that may be caused by a general medical or surgical illness [128]. Additionally, they do not facilitate the establishment of a therapeutic compromise between the patient’s treatment-related values and the goal of achieving the best treatment outcome. This oversight can lead to challenges in effectively addressing the complexities of patient care, particularly in situations where patients may struggle to articulate their values or make informed decisions due to their medical condition. In such cases, it is crucial for healthcare providers to adopt a more flexible and empathetic approach that considers the patient’s psychological state and fosters open communication. This can help ensure that treatment plans are not only aligned with clinical goals but also respect and incorporate the patient’s individual values and preferences, ultimately leading to better health outcomes and patient satisfaction.

The concept of illness-induced psychological regression is rooted in Freudian theory, which provides a framework for understanding how personality is structured and how it can be affected by stressors such as illness. According to Freud, personality consists of enduring patterns of behaviour that reflect an individual’s values, belief systems, personal goals, standards, and their understanding of the external world [129, 130].

Freud’s model of personality includes three key components: Id, Ego, and Super Ego [131]. The Id is the most primitive part of the personality, driven by basic instincts and desires. It operates on the pleasure principle, seeking immediate gratification without regard for reality or social norms. The Ego develops to mediate between the desires of the Id and the constraints of reality and societal expectations. It functions to regulate instinctual drives, perform reality testing, make judgments, and maintain a sense of self and the external world. The Ego employs various defense mechanisms to manage anxiety arising from conflicts between the Id and the Superego. The Superego represents internalised societal norms and moral values. It acts as a conscience, guiding behaviour according to what is considered right or wrong based on cultural standards.

Defence mechanisms are intra-psychic processes and behaviours that help reconcile internal drives with external demands [131]. They can be categorised into a maturational hierarchy, which includes psychotic defenses, immature or borderline defenses, neurotic defenses, and mature or normal defences [132, 133, 134].

Psychotic defences: This category includes mechanisms such as psychotic denial, psychotic distortion, and delusional distortion. Immature defenses: These consist of behaviours like passive aggression, acting out, dissociation, projection, autistic fantasy, devaluation, idealisation, and splitting. Neurotic defences: This level encompasses mechanisms such as intellectualisation, isolation, repression, reaction formation, displacement, somatisation, undoing, and rationalisation. Mature defenses: The most adaptive mechanisms include suppression, altruism, humour, and sublimation [135].

A critical consideration for clinical practice is that patients may experience APR under the stress of a general medical or surgical illness, potentially leading them to revert to less mature defense mechanisms and even develop borderline personality traits. Those with borderline personality traits often fluctuate between narcissistic tendencies, where they expect to be treated as significant individuals, and masochistic behaviours, which involve viewing themselves as deeply inadequate and worthless [136]. Patients with APR may also exhibit paranoid traits, believing that others wish to harm them. Additionally, they might interpret treatment suggestions from clinicians as threats to their self-identity, employing defence mechanisms that distort their self-image and ultimately decline treatment. These patients frequently struggle with trust in authority figures and are prone to misinterpret environmental cues. To address these issues effectively, clinicians should be attuned to the typical disruptive behaviours associated with borderline personality traits and adopt an interpretive model of the doctor-patient relationship as a foundational strategy. Cognitive Behavioural Therapy (CBT) is essential in helping these patients alter their dysfunctional thoughts and behaviours [136].

According to a psychodynamic theory proposed by Kernberg O. (1996) [135], individuals are categorised based on their Ego functions—specifically, reality testing, the status of identity diffusion, and the predominant level of defensive operations—into three categories: psychotic, borderline, and neurotic personality organisations [135]. Individuals with borderline personality organisation exhibit intact reality testing, significant identity diffusion, and employ primitive psychological defences such as projection, denial, distortion, and splitting. Kernberg’s theory [135] of personality organisation does not fully account for psychodynamic changes in patients experiencing APR, particularly because such patients often do not exhibit impairment in identity diffusion. Additionally, there is a contextual validity concern regarding Kernberg’s reality testing scale [128], as it does not enable clinicians to assess a patient’s decision-making capacity—specifically, their ability to make choices, understand, appreciate, and reason. This limitation affects the foundation of an effective doctor-patient relationship. Conversely, the broader concept of mental competence, which encompasses insight and judgement, extends beyond Kernberg’s notion of reality testing and carries significant medico-legal implications for the doctor-patient relationship [128].

Clinicians caring for patients who refuse treatment must consider not only the patients’ mental competence and treatment preferences but also the potential for illness-induced APR. To achieve optimal medical outcomes in patients who demonstrate the Actual Understanding test of mental competence, clinicians should adopt a deliberate model of the medical professional relationship. For patients who meet the Understanding test of mental competence and wish to engage with their health-related values, physicians are advised to implement an interpretive model of the doctor-patient relationship [128, 137]. In cases where mentally competent patients experience APR due to illness, initiating treatment with the interpretive model, combined with CBT, can be effective in addressing and modifying treatment-rejecting behaviours.

Managing mentally competent JW patients with ATAAD who refuse blood transfusions raises significant ethical challenges. Clinicians may struggle to align with the patients’ health-related values. The interpretive model of the clinician-patient relationship emphasizes honouring the values of JW patients, even if their choices might negatively impact medical results. This model respects and validates the religious beliefs and health preferences of JW patients.

Additionally, healthcare resource constraints may hinder the ability to provide high-dependency care, such as that found in an intensive care unit (ICU) or high-dependency unit (HDU). In such circumstances, severely anaemic JW patients who refuse blood transfusion may find themselves competing with other critically ill patients for limited ICU or HDU beds [138]. Here, the ethical principle of patient autonomy conflicts with the principle of formal justice, which asserts that individuals in similar situations should be treated equally [139].

Performing surgery on a JW patient with ATAAD without blood transfusions presents significant risks, primarily due to the potential for exsanguination. However, the approach to blood product acceptance among JW patients varies widely [116]. Some patients may refuse all blood products and cell saver techniques, while others might accept certain blood components such as platelets, albumin, or topical haemostatic agents, but decline others like ARBC transfusions or clotting factors like factor VII, prothrombin complex concentrate.

The interpretive-deliberative model of communication is an effective approach for managing complex cases involving JW patients refusing blood transfusions [140]. This model involves a multidisciplinary team—including surgeons, anaesthetists, and haematologists—working collaboratively with the mentally competent patient. The interpretive component centres on understanding and respecting the patient’s beliefs, whilst the deliberative component entails providing comprehensive, informed discussions regarding medical risks and exploring strategies to optimise surgical outcomes. The primary aim is to establish a therapeutic compromise that balances the patient’s preferences concerning blood transfusions with the imperative to minimise operative risks. This approach promotes shared decision-making, upholds patient autonomy, and endeavours to achieve the most favourable surgical outcome through collaborative engagement.

The ethical and clinical challenges inherent in the physician-patient relationship are perhaps most evident in cases where patients’ beliefs conflict with standard medical practices, such as the refusal of blood transfusions by JWs. Addressing these complex situations requires a deep understanding of ethical models, psychological factors, and the potential for illness-induced APR. The interpretive-deliberative model offers a promising framework for navigating these difficult decisions, fostering collaboration, and promoting shared decision-making. By prioritising open communication, respecting patient autonomy, and striving for a therapeutic compromise, clinicians can navigate these challenging scenarios with both ethical integrity and a commitment to achieving the best possible outcomes.

### 3.12 Innovative Approaches in Surgical and Endovascular Management of Extensive ATAAD

In patients with ATAAD involving a dissection flap extending through the arch into the descending thoracic aorta, an extended aortic repair strategy may be employed. This approach can include simultaneous aortic arch replacement, such as the “branch-first” technique, combined with antegrade stenting of the proximal descending thoracic aorta using a frozen elephant trunk (FET) technique [62, 141]. Alternatively, a hybrid approach performed simultaneously or on a sequential basis may be adopted, involving aortic root reconstruction and arch replacement, followed by thoracic or thoraco-abdominal endovascular aortic repair (TEVAR) [76, 141]. The use of a FET graft in surgical repair of ATAAD is particularly considered when there is a large tear at the distal arch or proximal descending aorta, or in cases of a full circumferential dissection at the level of the distal arch [62]. This strategy aims to stabilise the dissected segments, address primary entry tears, and reduce the risk of late complications such as re-dissection or aneurysm formation.

The “branch-first” procedure for aortic arch replacement involves a series of carefully coordinated steps designed to optimise cerebral and systemic perfusion during complex aortic surgery [62]. The procedure commences with arch debranching performed on a beating heart using a modified trifurcation arch graft equipped with a side-arm perfusion port (TAPP graft, Vascutek Ltd., Renfrewshire, Scotland, UK), while the patient is maintained on full-flow CPB. Subsequently, the aortic root is reconstructed under cardioplegic arrest through techniques such as valve resuspension, reimplantation, remodelling, or a Bentall’s procedure, depending on the underlying pathology. Distal circulatory arrest is then initiated under moderate hypothermia, with cerebral perfusion maintained via the TAPP graft at approximately 1 litre per minute. The aorta is then transected proximal to the origin of the left subclavian artery. A frozen elephant trunk (FET) graft, such as Thoraflex with an “Ante-flo” arm, is introduced and deployed into the proximal descending aorta, with the distal anastomosis constructed between the graft and the native aorta. The “Ante-flo” arm, connected to CPB, allows immediate antegrade systemic perfusion following cross-clamping of the non-stented proximal aortic segment. Finally, a straightforward proximal anastomosis is performed, followed by an end-to-side anastomosis of the TAPP graft to the ascending aorta graft. This comprehensive approach ensures continuous cerebral and systemic perfusion, streamlines the complex reconstruction process, and aims to improve surgical outcomes in extensive aortic arch pathology.

TEVAR for ATAAD is considered in specific clinical scenarios. These include cases where the entry tear is in the descending aorta or distal arch, in patients presenting with limb or visceral ischaemia associated with malperfusion, or in instances of chronic dissection or an aneurysm of the descending aorta [62, 76, 142].

The procedure involves the deployment of a covered stent graft, such as the Zenith TX2 TAA Endovascular Graft (Cook Medical, Bloomington, IN, USA), introduced and positioned within a Dacron proximal landing zone [62]. Typically, the stent is oversized by 10–15% relative to the surgically inserted Dacron graft. The initial covered stent is deployed so that its distal end lies between the junction of the upper and middle thirds of the descending thoracic aorta. Additional covered stents may be used as required, extending down to the diaphragm; however, extension to the diaphragm is generally avoided to reduce the risk of paraplegia. Subsequently, the remaining thoracic and abdominal aorta are lined with bare metal uncovered stent grafts, such as the Zenith Dissection Endovascular Stent (Cook Medical Inc., Bloomington, IN, USA). These bare metal stents are positioned with a proximal landing zone 1–2 cm inside the covered stent and deployed sequentially down the true lumen to the aortic bifurcation. An angioplasty balloon (e.g., Coda Balloon Catheter, Cook Medical Inc., Bloomington, IN, USA) is then used to sequentially expand the bare metal stents and rupture the septum between the true and false lumens, thereby creating a single, unified aortic channel. This process typically results in the realignment of branch vessels within the true lumen; however, if this does not occur, additional stent grafting may be necessary to restore flow in visceral vessels [62].

### 3.13 Postoperative Monitoring and Management of Residual Aortic Disease After Surgical Repair of ATAAD

Following surgical repair of ATAAD, patients with residual aortic disease require a structured surveillance protocol to monitor for potential complications. Imaging with computed tomography (CT) or magnetic resonance imaging (MRI) is recommended before discharge from hospital, at 1 month, 6 months, and 12 months postoperatively. If the aorta remains stable during this period, annual imaging is generally advised to detect any late changes or disease progression [8].

Reoperation after ATAAD is recommended in cases where a chronic residual thoraco-abdominal aneurysm (TAA) has developed with a total aortic diameter of ≥55 mm (class 1 recommendation, level C evidence) [4, 143]. In patients with an intact descending TAA who possess risk factors for rupture—such as aneurysm growth of ≥0.5 cm per year, symptomatic aneurysm, Marfan syndrome, Loeys-Dietz syndrome, vascular Ehlers-Danlos syndrome, heritable thoracic aortic disease, saccular aneurysm, female sex, or infectious aneurysm—repair may be considered at a diameter of less than 55 mm (class 2a recommendation, level C evidence) [4, 143]. Other indications for reoperation after ATAAD include the presence of aortic anastomotic pseudoaneurysms, progressive aortic regurgitation, and graft infection [144].

In the context of TEVAR, false-lumen thrombosis occurs in approximately 91% of cases with extent 3A dissection (dissection flap extending above the diaphragm) and 62% of extent 3B cases (dissection flap extending below the diaphragm). However, reintervention rates after TEVAR range from 15% to 26% at 5 years, largely depending on the extent and progression of the dissection [4].

Routine surveillance after endovascular repair aims to identify issues such as endoleaks, sac growth, endograft migration, or endograft failure. Typical surveillance intervals after TEVAR are at 1 month, 6 months, and 12 months and yearly thereafter [8]. CT aortography remains the gold standard for follow-up imaging after TEVAR due to its high diagnostic accuracy. However, it involves exposure to ionising radiation and iodinated contrast, which can be nephrotoxic. Duplex ultrasound is specific for detecting endoleaks but limited in assessing stent migration, fracture, or noncontiguous aneurysms. MRI offers high accuracy for endoleak detection but requires a plain abdominal radiograph to evaluate for stent fracture, as MRI cannot reliably visualise metallic stent components.

### 3.14 Strengths and Limitations of Current Research on ATAAD

The main strengths of this study include its systematic presentation of knowledge regarding the morphology, taxonomy, and socioeconomic factors associated with ATAAD. It also elucidates the pathophysiological mechanisms of ischaemia and haemorrhage and their impact on patient outcomes. Additionally, the review introduces a novel, interpretive-deliberate model of the doctor-patient relationship, particularly in cases where patients refuse blood transfusions. However, the study has several limitations. A primary limitation is the small number of available studies examining the relationship between the duration of global myocardial ischaemia, the depth of hypothermic circulatory arrest, and outcomes of ATAAD repair. There is also a notable lack of cohort studies involving JW patients with ATAAD who refuse blood transfusions. The mortality risks associated with untransfused severe anaemia were estimated from a heterogeneous group of JWs admitted with various medical and surgical conditions, which may limit the generalizability of these findings. Furthermore, the Auckland and Hamilton Anaemia Mortality Risk Scores have not been validated in other studies, which may affect their applicability and reliability in different clinical settings.

## 4. Conclusions

This review indicates that, although considerable advancements have been achieved in evaluating in-hospital mortality among patients with ATAAD, as well as in improving doctor-patient communication, refining anesthetic and perfusion techniques, and enhancing surgical management, additional research is necessary to confirm the effectiveness of these strategies.
